# δ-Tocotrienol and quercetin reduce serum levels of nitric oxide and lipid parameters in female chickens

**DOI:** 10.1186/1476-511X-10-39

**Published:** 2011-02-28

**Authors:** Asaf A Qureshi, Julia C Reis, Nilofer Qureshi, Christopher J Papasian, David C Morrison, Daniel M Schaefer

**Affiliations:** 1 Department of Basic Medical Sciences, University of Missouri-Kansas City, 2411 Holmes Street, Kansas City, MO 64108, USA; 2 Department of Pharmacology/Toxicology, School of Pharmacy, 2464 Charlotte Street, Kansas City, MO 64108, USA; 3 Department of Animal Sciences, University of Wisconsin, Madison, WI. 53706, USA

## Abstract

**Background:**

Chronic, low-grade inflammation provides a link between normal ageing and the pathogenesis of age-related diseases. A series of *in vitro *tests confirmed the strong anti-inflammatory activities of known inhibitors of NF-κB activation (δ-tocotrienol, quercetin, riboflavin, (-) Corey lactone, amiloride, and dexamethasone). δ-Tocotrienol also suppresses β-hydroxy-β-methylglutaryl coenzyme A (HMG-CoA) reductase activity (the rate-limiting step in *de novo *cholesterol synthesis), and concomitantly lowers serum total and LDL cholesterol levels. We evaluated these compounds in an avian model anticipating that a dietary additive combining δ-tocotrienol with quercetin, riboflavin, (-) Corey lactone, amiloride, or dexamethasone would yield greater reductions in serum levels of total cholesterol, LDL-cholesterol and inflammatory markers (tumor necrosis factor-α [TNF-α], and nitric oxide [NO]), than that attained with the individual compounds.

**Results:**

The present results showed that supplementation of control diets with all compounds tested except riboflavin, (-) Corey lactone, and dexamethasone produced small but significant reductions in body weight gains as compared to control. (-) Corey lactone and riboflavin did not significantly impact body weight gains. Dexamethasone significantly and markedly reduced weight gain (>75%) compared to control. The serum levels of TNF-α and NO were decreased 61% - 84% (*P *< 0.001), and 14% - 67%, respectively, in chickens fed diets supplemented with δ-tocotrienol, quercetin, riboflavin, (-) Corey lactone, amiloride, or dexamethasone as compared to controls. Significant decreases in the levels of serum total and LDL-cholesterol were attained with δ-tocotrienol, quercetin, riboflavin and (-) Corey lactone (13% - 57%; *P *< 0.05), whereas, these levels were 2-fold higher in dexamethasone treated chickens as compared to controls. Parallel responses on hepatic lipid infiltration were confirmed by histological analyses. Treatments combining δ-tocotrienol with the other compounds yielded values that were lower than individual values attained with either δ-tocotrienol or the second compound. Exceptions were the significantly lower total and LDL cholesterol and triglyceride values attained with the δ-tocotrienol/(-) Corey lactone treatment and the significantly lower triglyceride value attained with the δ-tocotrienol/riboflavin treatment. δ-Tocotrienol attenuated the lipid-elevating impact of dexamethasone and potentiated the triglyceride lowering impact of riboflavin. Microarray analyses of liver samples identified 62 genes whose expressions were either up-regulated or down-regulated by all compounds suggesting common impact on serum TNF-α and NO levels. The microarray analyses further identified 41 genes whose expression was differentially impacted by the compounds shown to lower serum lipid levels and dexamethasone, associated with markedly elevated serum lipids.

**Conclusions:**

This is the first report describing the anti-inflammatory effects of δ-tocotrienol, quercetin, riboflavin, (-) Corey lactone, amiloride, and dexamethasone on serum TNF-δ and NO levels. Serum TNF-δ levels were decreased by >60% by each of the experimental compounds. Additionally, all the treatments except with dexamethasone, resulted in lower serum total cholesterol, LDL-cholesterol and triglyceride levels. The impact of above mentioned compounds on the factors evaluated herein was increased when combined with δ-tocotrienol.

## Background

Inflammatory responses to a wide variety of stimuli are largely attributable to up-regulation of the pro-inflammatory transcription nuclear factor kappaB (NF-κB). Specifically, reactive oxygen species (ROS) up-regulate the pro-inflammatory NF-κB transcription factor. The increased transport of NF-κB to the cell nucleus enhances expression of numerous genes encoding proteins that contribute to the inflammatory process, including inducible nitric oxide synthase (iNOS), cyclooxygenase-2 (COX-2), tumor necrosis factors (TNF-α, TNF-β), interleukins (IL-1, IL-6), chemokines (IL-8, MCP1, and MIP1α), activator protein-1 (AP-1) and adhesion factors (ICAM, and VCAM). Several of the proteins encoded by genes that are up-regulated by NF-κB are also potent NF-κB activators, thereby, forming an auto-activating loop. With ageing, the capacity to maintain a proper redox balance weakens with a concomitant up-regulation of NF-κB [1,2]. Age-associated activation of NF-κB has the expected effect of increasing serum levels of TNF-α and nitric oxide (NO), and increased NO production has been observed during senescence [1-3]. These changes appear to be important as there is increasing evidence to support the concept that chronic, low-grade, and systemic inflammation contributes to the development of metabolic syndrome, dementia, cancer, atherosclerosis, osteoporosis, and other age-related diseases [1,2].

NF-κB is normally activated by degradation of inhibitory kappaB (IκB). When this occurs, NF-κB translocates to the nucleus and binds to specific promoter regions of genes encoding pro-inflammatory proteins. Over the past decade, investigators have identified a number of compounds that selectively interfere with the NF-κB pathway. Several of these compounds are plant-derived antioxidants [4]. Quercetin and other flavonoids [5-10], and tocotrienols [11-18] suppress protein kinase-mediated degradation of inhibitory kappaB (IκB). This prevents NF-κB activation, and the corresponding increase in production of various inflammatory proteins [7,9]. These ubiquitous plant constituents have been reported to suppress the progression of age-related diseases [19].

Quercetin and related flavonoids improve mental acuity [20], promote bone health [21], and attenuate carcinogenesis [7,22,23], development of atherosclerotic plaque [22-24], and diabetes [25]. Tocols, principally the tocotrienols, attenuate diabetic neuropathy [16], slow diabetes-associated cognitive decline [17], protect neurons from glutamate toxicity [26], and support bone formation [27]. The impact of α-, β-, γ- and δ-tocotrienols, the naturally-occurring farnesylated (unsaturated side-chain) analogs of α-, β-, γ- and δ-tocopherols, on atherosclerosis [28-34] and cancer [35-39] has been reported and reviewed by several investigators (30-39). The antioxidant activity associated with all tocols suppresses protein kinase-mediated inhibitory kappaB (IκB) degradation and subsequent NF-κB activation. Tocotrienols appear to be unique among the tocols, as they suppress hepatic HMG-CoA reductase activity and the synthesis of mevalonate-derived products through a yet to be defined [40] post-transcriptional mechanism [41]. As a consequence, tocotrienols uniquely lower serum total cholesterol and LDL-cholesterol levels [31-34,42-46]. Moreover, when evaluated *in vitro*, tocotrienols suppress the synthesis of inflammatory cytokines with greater potency than the tocopherols [11-17,47].

As delineated above, tocopherols and tocotrienols suppress protein kinase-mediated IκB degradation and concomitant NF-κB activation initiated by reactive oxygen species (ROS). The tocotrienols additionally suppress TNF-α stimulated NF-κB activation. This suppression is reversed by treating with mevalonate and other products of HMG-CoA reductase activity [12,14]. This reversal is consistent with findings that Rho GTPases involved in the regulation of NF-κB require a post-translational modification, specifically prenylation, in order to be active [48].

**Figure 1:**
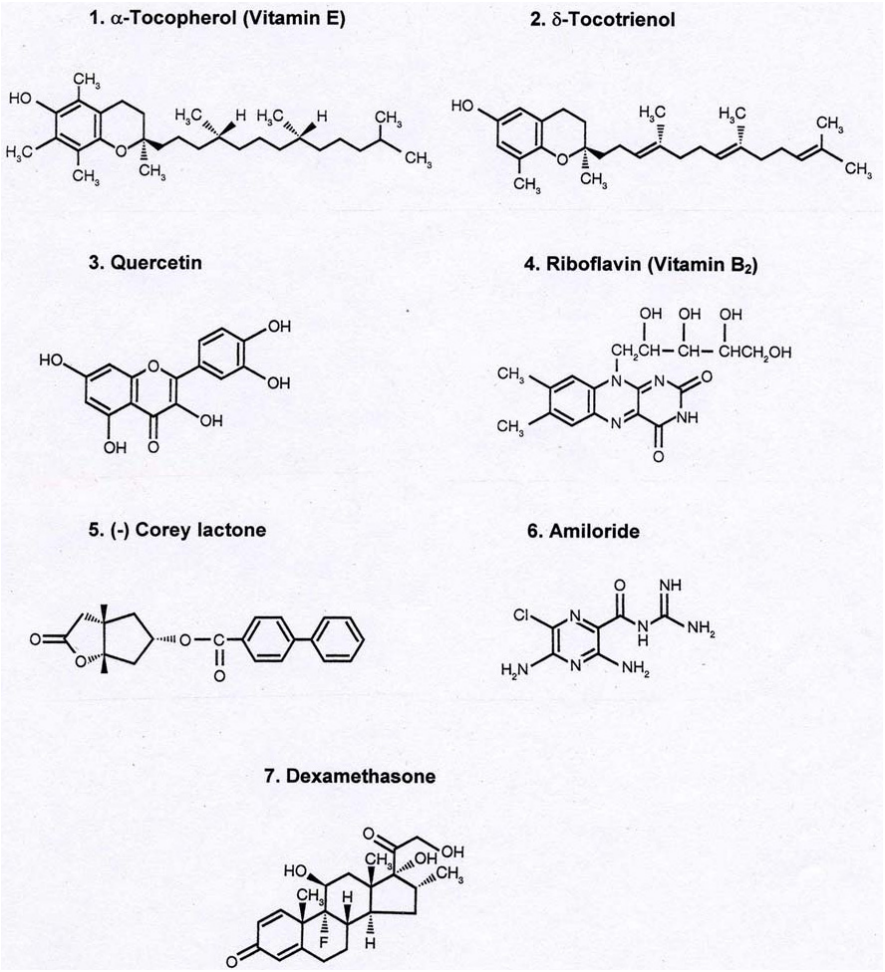
**Chemical structures of various compounds used in this study**.

δ-Tocotrienol, quercetin, riboflavin, amiloride, dexamethasone and (-) Corey lactone (an intermediate in prostanoid synthesis), are all FDA approved compounds and have been used by humans for several decades without any alarming adverse affects. All these compounds inhibit lipopolysaccharide (LPS)-induced TNF-α and iNOS gene expression in macrophages prepared from RAW 264.7 cells, BALB/c and C57BL/6 mice [unpublished results]. These key mediators of inflammation are up-regulated during the ageing process [1-3]. Therefore, we now evaluate the impact of the aforementioned compounds on serum NO and TNF-α levels. We tested the hypothesis that a dietary blend consisting of δ-tocotrienol plus quercetin, riboflavin, (-) Corey lactone, amiloride, or dexamethasone (Figure 1) would be more effective than the individual compounds in lowering serum NO and TNF-α levels. As described earlier, δ-tocotrienol additionally suppresses cholesterol synthesis and concomitantly lowers serum total cholesterol and LDL-cholesterol levels in various experimental animal models and humans [41-45]. We focused on the impact of these compounds on serum levels of TNF-α, NO, total cholesterol, LDL-cholesterol, and triglyceride in young female chickens. This avian model, chickens [49], differing from the widely employed rodent model [50], closely reflects human lipid metabolism.

## Materials and methods

### Materials

Sources of all chemicals, substrates, and diagnostic kits have been identified previously [51]. Chemicals and solvents were of analytical grade. Riboflavin, (-) Corey lactone = 4-phenylbenzoate alcohol = (3aR,4S,5R,6aS)-hexahydro-4-hydroxymethyl-5-(4-phenylbenzoyloxy) cyclopenta [b] furon-2-one], amiloride, and dexamethasone were purchased from Sigma-Aldrich (St. Louis, MO, USA). Quercetin was purchased from Alfa Aesar (Johnson Matthey Co. Lancastor, UK); lipid extracts of annatto seeds consisting of 50% δ-tocotrienol were purchased from American River Nutrition (Hadley, MA, USA).

### Isolation of δ-tocotrienol from lipid extract of annatto seeds

Silica gel (Merck, 230-400 mesh, 60 Å, 500 g) suspended in 1000 mL of hexane was poured into a 2-L glass funnel with a fritted disk. The gel was washed with 2 L of hexane prior to being loaded with 100 g of the 50% δ-tocotrienol fraction of annatto seed in 200 mL of hexane. Contaminants were washed with two liters of 10% diethyl ether in hexane. δ-Tocotrienol was then eluted with 30% diethyl ether in hexane (2 L). The eluted fraction, evaporated under vacuum at 50°C, yielded 36 g of δ-tocotrienol. The purity (98%) was determined by high pressure liquid chromatography (HPLC) as described [43].

### Diets, experimental conditions

White Leghorn one-day-old female chicken and diet ingredients were supplied by the Poultry Research Laboratory, University of Wisconsin, Madison, WI, USA. The diet consists of corn (8.8% protein, 615 g), soybean meal (44% protein, 335 g), tocols-stripped corn oil (10 g), calcium carbonate (10 g), dicalcium phosphate (20 g), iodized salt (5 g), and mineral and vitamin mixtures (2.5 g of each), which provided per kg feed: zinc sulfate.H_2_O, 110 mg; manganese sulfate.5H_2_O, 70 mg; ferric citrate.H_2_O, 500 mg; copper sulfate.5H_2_O, 16 mg; sodium selenite, 0.2 mg; d,l-methionine, 2.5 mg; choline chloride (50%), 1.5 g; ethoxyquin, 125 mg; thiamine.HCl, 1.8 mg; vitamin A, 1500 units; vitamin D3, 400 units; vitamin E, 10 mg; riboflavin, 3.6 mg; calcium pantothenate, 10 mg; niacin 25 mg; pyridoxine.HCl, 3 mg; folacin, 0.55 mg; biotin, 0.15 mg; vitamin B_12 _0.01 mg; and vitamin K, 0.55 mg. Corn additionally provided an estimated 2 mg/kg tocols consisting of 12% α-tocopherol, 3% α-tocotrienol, 7% β-tocotrienol, 70% γ-tocopherol, 6% γ-tocotrienol, 2% δ-tocopherol, per g [52].

Day-old female chickens were fed a corn-soy meal diet for one week before being randomly assigned to one of 24 groups. Three control groups (*n *= 18, housed 6/cage/group) was fed the aforementioned commercial diet for four weeks. One experimental group (*n *= 6) was fed a diet supplemented with 50 ppm δ-tocotrienol (125 μM/kg); twenty experimental groups were fed a diet supplemented with 25 or 50 ppm quercetin (74, or 148 μM/kg), 25 or 50 ppm riboflavin (66.5 or 133 μM/kg), 25 or 50 ppm (-) Corey lactone (71 or 142 μM/kg), 5 or 10 ppm amiloride (16.5 or 33 μM/kg), or 0.5 or 1 ppm dexamethasone (1.27 or 2.55 μM/kg) with or without 50 ppm δ-tocotrienol (125 μM/kg) for four weeks.

Dietary concentrations of the aforementioned compounds were derived from our preliminary studies of their effects on TNF-α, and nitric oxide production by RAW 264.7 cells and by macrophages prepared from several strains of mice [unpublished results]. All treatment compounds except dexamethasone were dissolved in 50 mL 95% ethanol; dexamethasone was dissolved in 50 mL deionized water (100°C). The compounds were mixed with the commercial diet (5 kg) in Food Mixer for 30 min to eliminate the solvent. The experimental diets were kept at room temperature throughout the feeding period.

Groups were housed in a single brooder with 24 h light and free access to water and diet. Chickens were weighed at the start and end of the trial. At the end of 4-week feeding period, the birds were fasted for 12 h prior to sacrifice to facilitate chylomicron and very-low-density lipoprotein (VLDL) clearance. The chickens were sacrificed by severing their carotid arteries, rather than gas euthanasia, in order to keep the blood (serum) composition intact for determining TNF-α and NO levels. Livers were collected, weighed, and stored at -70°C; a small portion of each liver was stored in 10% formalin and stored at -70°C pending histological analyses. The blood samples were incubated at 37°C for 20 min and centrifuged at 10,000 × g for 20 min to collect sera, which were held at -70°C, pending analyses. The protocol was reviewed and approved by the University of Wisconsin-Madison College of Agriculture and Life Sciences Animal Care and Use Committee. The study was carried out under a FDA approved IND number 36906.

### General biochemical methods and techniques

#### Assays of serum lipid parameters

The analyses of coded samples were performed at the University of Missouri, Kansas City, School of Medicine, MO, USA. Serum cholesterol and triglyceride levels were estimated using Kits # 352 and 336, respectively, purchased from Sigma Chemical Co. St. Louis, MO, USA. LDL-cholesterol was precipitated from 200 μL of serum with 25 μL of a mixture of 9.7 mM phosphotungstic acid and 0.4 M MgCl_2_. The preparation was mixed for 10 min at room temperature and then centrifuged at 12,000 × g for 20 min. The supernatant was decanted and analyzed for HDL-cholesterol. The remaining precipitate was dissolved in 200 μL of 0.1 M sodium citrate and the level of LDL-cholesterol estimated as described for total cholesterol [51]. All assays for each treatment were carried out at the same time under similar conditions to minimize standard deviation.

### Measurement of TNF-α level in serum of 5-week-old female chickens

Levels of TNF-α in serum of chickens were determined by Quantikine M ELISA kit (R & D System, Minneapolis, MN, USA) according to manufacturer's instructions. The lower limit of detection for TNF-α in this method is approximately, 5.0 pg/mL [53].

### Measurement of nitric Oxide (NO) level in serum of 5-week-old female chickens

Production of NO in serum of chickens was determined by measuring the amount of nitrite, a stable metabolic product of nitric oxide as described previously [54]. The assay mixture consisted of medium (100 μL) and Griess reagent (100 μL) placed in round-bottom 96-well tissue culture plates (incubation time 30 min) and absorption was measured at 570 nm on a "Microplate Reader" (MR 5000; Dynatech Labs, Inc. USA). The amount of nitrite was determined by comparison of unknowns with a NaNO_2 _standard curve. The nitrite detection limit is 0.20 nM.

### Histological studies of liver samples of 5-week-old female chickens

Liver tissues, fixed in 10% formalin, were embedded with paraffin and cut in the sagital plane. The sections were stained with hematoxylin and eosin and examined by light microscopy, and were evaluated by two pathologists, each blinded to the treatments. A semi-quantitative evaluation of histological analyses of these liver samples was carried out according to published methods [55]. Mean scores were assigned to each sample, scored range 5 (presence of impact) to 40 (very severe impact). Sample with normal appearance received a score of "0". The means of assigned values for each group were determined, and based on these evaluations summary of each treatment were reported.

### Microarray data and pathway analyses of RNA of liver samples of 5-week-old female chickens

Small frozen sections, randomly collected from each liver within a treatment group, were pooled and the RNA was isolated and purified using an affinity resin column (RNeasy, Qiagen, Chatsworth, CA, USA), as described previously [56] and then analyzed at Mayo Clinic (Rochester, Minnesota, USA) using an Affymetrix Gene-Chip (chicken), and Expression array analysis of chicken [57]. Gene expression data were first imported in Genespring program (Agilento Palo Alto, CA, USA). The expression values of up-regulated genes showed positive numbers, whereas the down-regulated genes showed negative numbers. Various genes were identified by using GeneSifter software. A number of sets of cluster analyses of various ranges of genes sets (226, 465, 500, 1000, 1500 and 2000) were mapped to get quantitative data. The analysis of amiloride was not carried out due to published reports [58].

### Statistical analysis

Stat-View software (4.01) was used for the analyses of treatment-mediated effects (1992; Abacus Concepts, Berkeley, CA, USA). Treatment-mediated differences in serum lipid (total cholesterol, LDL-cholesterol, HDL-cholesterol, triglycerides), TNF-α and NO levels and weight (gain, liver weight, relative liver weight) were identified using a two-way analysis of variance (ANOVA). When the F test indicated a significant effect, the differences between the means were analyzed by Fisher's Protected Least Significance Difference (LSD) test. Data are reported as mean ± SD in the text and tables. The statistical significance level was set at 5% (*P *< 0.05).

## Results

The impact of 2-concentrations of quercetin, riboflavin, (-) Corey lactone (25 and 50 ppm), amiloride (5 and 10 ppm) and dexamethasone (0.5 and 1 ppm) with and without δ-tocotrienol (50 ppm) on inflammatory markers and lipid parameters were studied in 5-week-old female chickens. On completion of the four-week study the data revealed that varying the concentration of these compounds failed to produce significant differences. Consequently, we present results only for the data reflecting results obtained by feeding the higher concentrations. There were 24 groups in the study, including 3 control groups. The birds of control group # 1 were sacrificed in the beginning, # 2 in the middle (after group # 12) and birds of third control group (# 3) were sacrificed after group # 23. The values presented for the control group represent the average of three control group values (*n *= 18).

### Effect of various compounds on weight gain and relative liver weight gain of 5-week-old female chickens

Each of the compounds, with the exception of (-) Corey lactone and riboflavin, significantly lowered body weight gain (Table 1). Treatments combining δ-tocotrienol and (-) Corey lactone or amiloride significantly increased body weight gain when compared to the effect of the individual compound and the control. The opposite response, a significant lowering of body weight gain, was detected for a treatment combining δ-tocotrienol and dexamethasone; a similar trend, though not significant, was detected for the treatment combining δ-tocotrienol and riboflavin. Treatment-mediated impacts on feed efficiency (grams feed/gram weight gain) and liver weight paralleled those noted for weight gain (Table 1). Riboflavin, amiloride, and to a much greater extent, dexamethasone, significantly increased relative liver weights when compared to that of the control group. δ-Tocotrienol attenuated the impact of amiloride on relative liver weight as shown in Table 1.

**Table 1:** Effects of various compounds on body weight gain and relative liver weight of 5-week-old female chickens^1^.

	Nutritional state	Body weight	Liver weight/100 g
		**Gain (g)**	**Body weight**

1	Control Diet (CD)^2^	240.12 ± 11.37^bc^	2.41 ± 0.29^ef^
2	CD + δ-Tocotrienol (50 ppm)	218.83 ± 8.16^e^	2.42 ± 0.19^ef^
3	CD + Quercetin (50 ppm)	225.50 ± 5.96^de^	2.35 ±0.23^ef^
4	CD + Riboflavin (50 ppm)	231.17 ± 7.52^cd^	2.61 ± 0.18^cd^
5	CD + (-) Corey lactone (50 ppm)	244.00 ± 5.02^b^	2.60 ± 0.18^cde^
6	CD + Amiloride (10 ppm)	230.33 ± 11.91^de^	2.80 ± 0.25^c^
7	CD + Dexamethasone (1.0 ppm)	57.00 ± 8.41^f^	4.44 ± 0.21^b^
			
	**δ-Tocotrienol (50 ppm) blend**^**3**^		
			
8	CD + δ-T3 + Quercetin (50 ppm)	243.50 ± 10.0^b^	2.55 ± 0.23^cdef^
9	CD + δ-T3 + Riboflavin (50 ppm)	228.50 ± 13.50^de^	2.75 ± 0.20^cd^
10	CD + δ-T3 + (-) Corey lactone (50 ppm)	266.83 ± 13.86^a^	2.48 ± 0.27^def^
11	CD + δ-T3 + Amiloride (10 ppm)	259.50 ± 4.18^a^	2.27 ± 0.14^f^
12	CD + δ-T3 + Dexamethasone (1.0 ppm)	39.33 ± 7.20^g^	5.75 ± 0.48^a^

^1^Feeding period was 4 weeks; Data expressed as means ± SD = 6 chickens per group.

^2^The value of control group was an average of 3 control groups.

^3^The diets of groups 8 - 12 were supplemented with δ-tocotrienol (50 ppm).

^a-g^Values in columns not sharing a common superscript letter are significantly different at *P *< 0.05.

**Figure 2:**
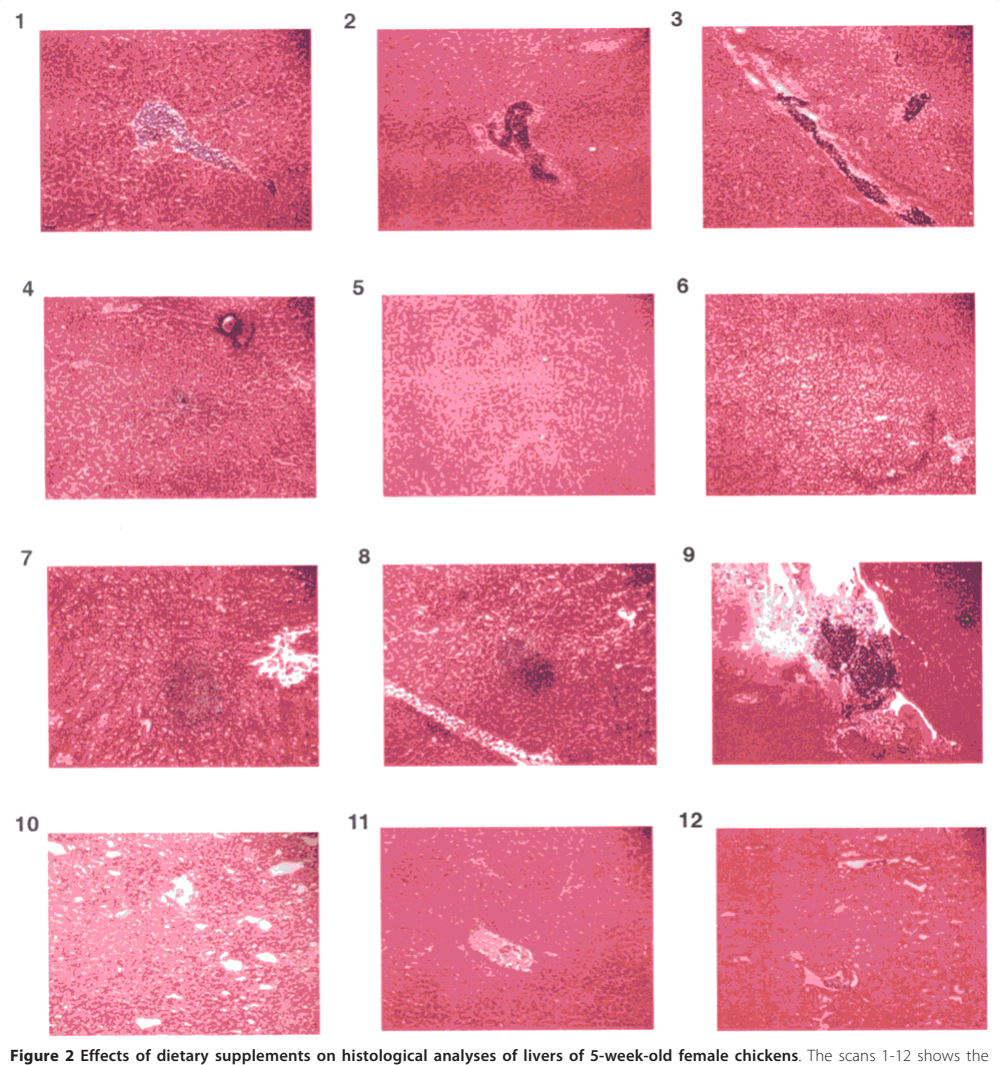
**Effects of dietary supplements on histological analyses of livers of 5-week-old female chickens**. The scans 1-12 shows the histological evaluation of representative liver sections from chickens treated with or without δ-tocotrienol with other compounds: 1. control diet; 2. δ-tocotrienol (δ-T3); 3. quercetin; 4. riboflavin; 5. (-) Corey lactone; 6. amiloride; 7. dexamethasone; 8. δ-T3 + quercetin; 9. δ-T3 + riboflavin; 10. δ-T3 + (-) Corey lactone; 11. δ-T3 + amiloride; 12. δ-T3 + dexamethasone.

The impacts of the treatments on liver slices (histological analyses) are recorded in Table 2. Severe chronic inflammation with mild fatty infiltration was recorded for the liver samples of control group. All individual treatments reduced the extent of inflammation (Table 2; Figure 2). Combining δ-tocotrienol with quercetin, riboflavin, (-) Corey lactone, or amiloride, yielded greater reductions in inflammation and fatty infiltration than the reductions achieved with individual compounds. Combining δ-tocotrienol with dexamethasone yielded a significant increase in relative liver weight compared to that of the dexamethasone group (Table 1), and livers were severely compromised as shown by histological analyses (Figure 2).

**Table 2:** Effects of δ-tocotrienol and various compounds on histological analyses of liver samples of 5-week-old female chickens^1^.

#	Nutritional state	Histological analyses of chicken liver samples
1	Control Diet (CD)	Severe chronic inflammation & mild fatty infiltration
2	CD + δ-Tocotrienol (50 ppm)^2^	Mild chronic inflammation
3	CD + Quercetin (50 ppm)	Moderate chronic inflammation & mild fatty infiltration
4	CD + Riboflavin (50 ppm)	Moderate chronic inflammation & mild fatty infiltration
5	CD + (-) Corey lactone (50 ppm)	Mild chronic inflammation & moderate fatty infiltration
6	CD + Amiloride (10 ppm)	Mild chronic inflammation & moderate fatty infiltration
7	CD + Dexamethasone (1.0 ppm)	Mild chronic inflammation & moderate fatty infiltration
		
	**δ-Tocotrienol (50 ppm) blend**^**2**^	
		
8	CD + δ-T3 + Quercetin (50 ppm)	Moderate chronic inflammation & mild fatty infiltration
9	CD + δ-T3 + Riboflavin (50 ppm)	Very mild chronic inflammation
10	CD + δ-T3 + (-) Corey lactone (50 ppm)	Very mild chronic inflammation & autolysis
11	CD + δ-T3 + Amiloride (10 ppm)	Very mild chronic inflammation
12	CD + δ-T3 + Dexamethasone (1.0 ppm)	Autolysis

^1^Feeding period was 4 wk; Data expressed as means ± SD = 6 chickens per group.

^2^The diets of groups 8 - 12 were supplemented with δ-tocotrienol (50 ppm).

### Effects of various compounds on serum levels of TNF-α and NO of 5-week-old female chickens

**Figure 3:**
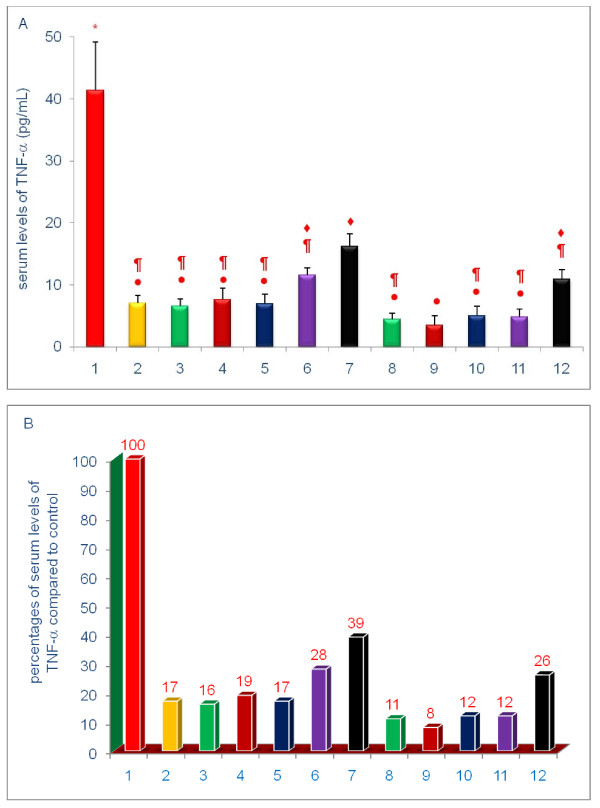
**Effects of dietary supplements on serum levels of TNF-α of 5-week-old female chickens**. The information regarding treatment groups were described in detail in the Methods section. Chickens were fed for 4 weeks. The diets of groups 8 - 12 were supplemented with δ-tocotrienol (50 ppm). Data expressed as means ± SD, *n *= 6 chickens per group. The value for the control group was the average of 3 control groups. Values in columns not sharing a common symbol were significantly different at *P *< 0.05. For each figure, 'A' shows the raw values for each of the treatments and control, and 'B' shows the percent change compared to control. The groups 1-12 correspond to: 1. control diet; 2; δ-tocotrienol (δ-T3); 3. quercetin; 4. riboflavin; 5. (-) Corey lactone; 6. amiloride; 7. dexamethasone; 8. δ-T3 + quercetin; 9. δ-T3 + riboflavin; 10. δ-T3 + (-) Corey lactone; 11. δ-T3 + amiloride; 12. δ-T3 + dexamethasone.

**Figure 4:**
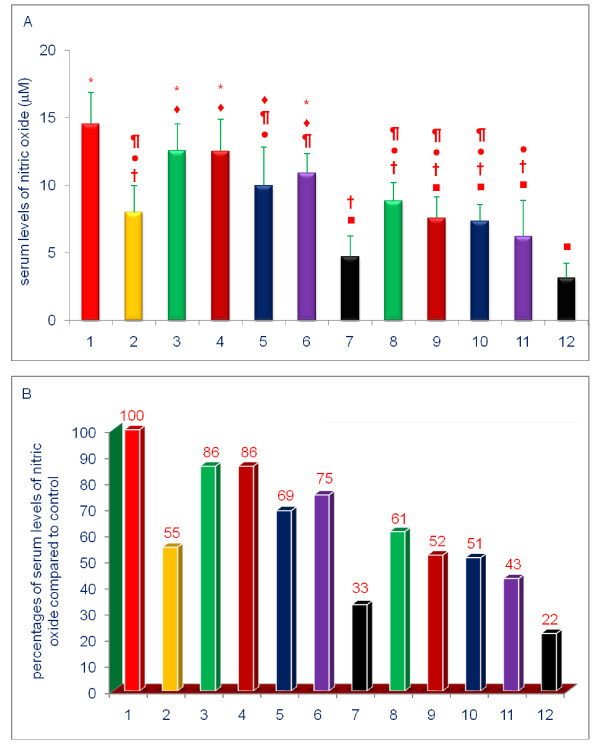
**Effects of dietary supplements on the serum levels of nitric oxide (NO) of 5-week-old female chickens**. Chickens were fed for 4 weeks. The diets of groups 8 - 12 were supplemented with δ-tocotrienol (50 ppm). Data expressed as means ± SD, *n *= 6 chickens per group. The value for the control group was the average of 3 control groups. Values in columns not sharing a common symbol were significantly different at *P *< 0.05. For each figure, 'A' shows the raw values for each of the treatments and control, and 'B' shows the percent change compared to control. The groups 1 - 12 correspond to: 1. control diet; 2; δ-tocotrienol (δ-T3); 3. quercetin; 4. riboflavin; 5. (-) Corey lactone; 6. amiloride; 7. dexamethasone; 8. δ-T3 + quercetin; 9. δ-T3 + riboflavin; 10. δ-T3 + (-) Corey lactone; 11. δ-T3 + amiloride; 12. δ-T3 + dexamethasone.

**Figure 5:**
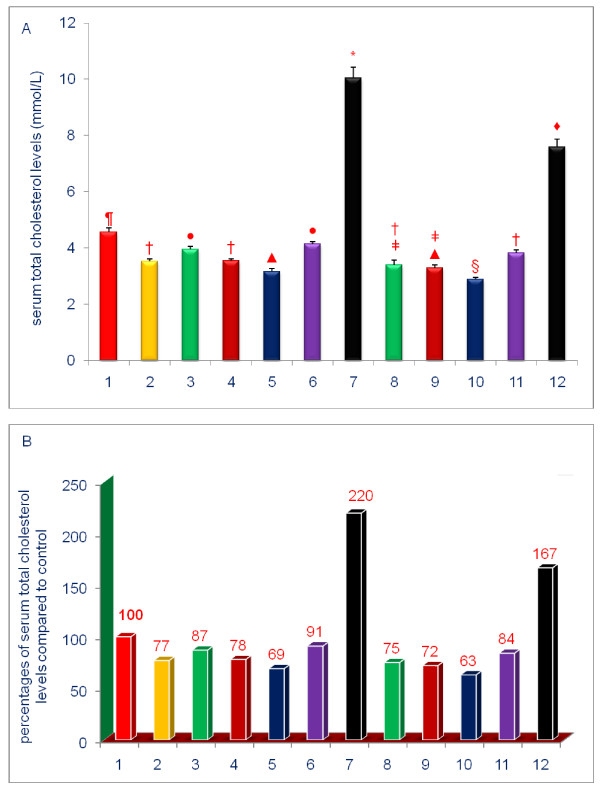
**Effects of dietary supplements on the serum levels of total cholesterol of 5-week-old female chickens**. Chickens were fed for 4 weeks. The diets of groups 8 - 12 were supplemented with δ-tocotrienol (50 ppm). Data expressed as means ± SD, *n *= 6 chickens per group. The results were reported in SI units (mmo/L). The value for the control group was the average of 3 control groups. Values in columns not sharing a common symbol were significantly different at *P *< 0.05. For each figure, 'A' shows the raw values for each of the treatments and control, and 'B' shows the percent change compared to control. The groups 1 - 12 correspond to: 1. control diet; 2; δ-tocotrienol (δ-T3); 3. quercetin; 4. riboflavin; 5. (-) Corey lactone; 6. amiloride; 7. dexamethasone; 8. δ-T3 + quercetin; 9. δ-T3 + riboflavin; 10. δ-T3 + (-) Corey lactone; 11. δ-T3 + amiloride; 12. δ-T3 + dexamethasone.

**Figure 6:**
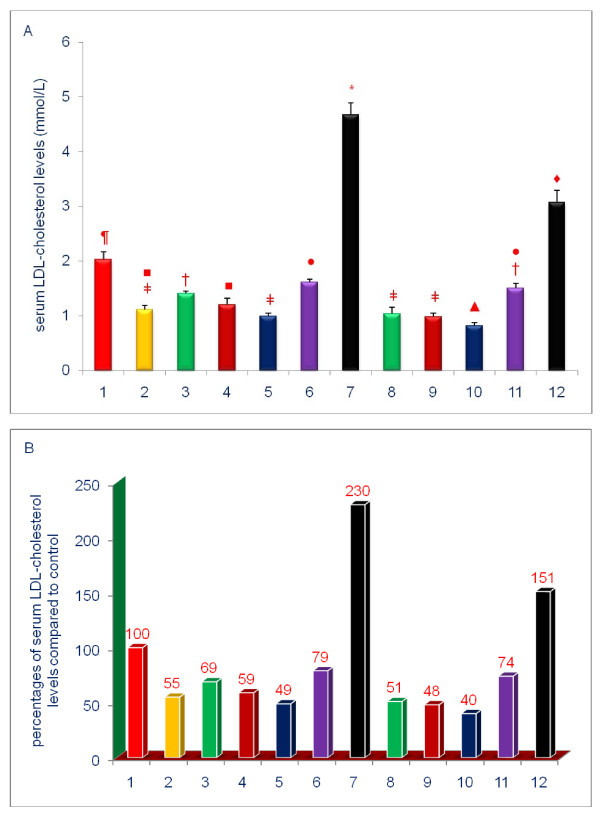
**Effects of dietary supplements on the serum levels of LDL-cholesterol of 5-week-old female chickens**. Chickens were fed for 4 weeks. The diets of groups 8 - 12 were supplemented with δ-tocotrienol (50 ppm). Data expressed as means ± SD, *n *= 6 chickens per group. The results were reported in SI units (mmo/L). The value for the control group was the average of 3 control groups. Values in columns not sharing a common symbol were significantly different at *P *< 0.05. For each figure, 'A' shows the raw values for each of the treatments and control, and 'B' shows the percent change compared to control. The groups 1 - 12 correspond to: 1. control diet; 2; δ-tocotrienol (δ-T3); 3. quercetin; 4. riboflavin; 5. (-) Corey lactone; 6. amiloride; 7. dexamethasone; 8. δ-T3 + quercetin; 9. δ-T3 + riboflavin; 10. δ-T3 + (-) Corey lactone; 11. δ-T3 + amiloride; 12. δ-T3 + dexamethasone.

**Figure 7:**
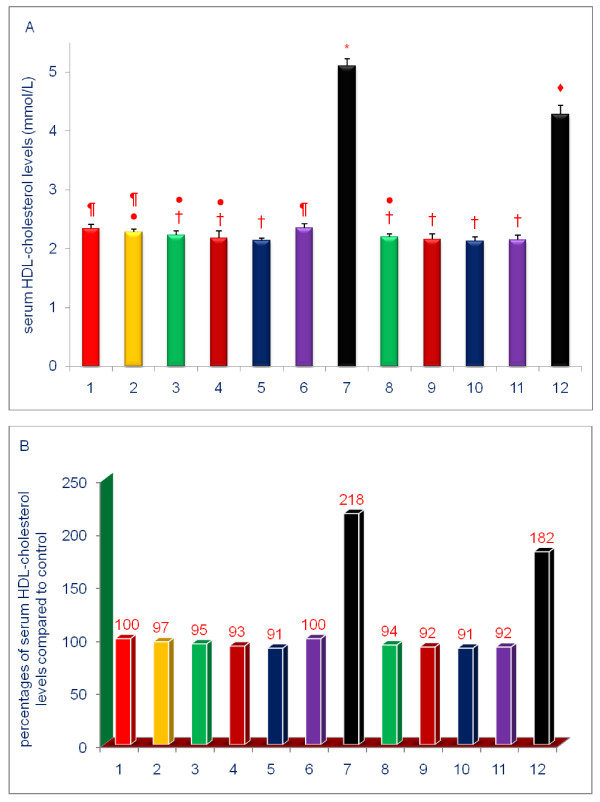
**Effects of dietary supplements on the serum levels of HDL-cholesterol of 5-week-old female chickens**. Chickens were fed for 4 weeks. The diets of groups 8 - 12 were supplemented with δ-tocotrienol (50 ppm). Data expressed as means ± SD, *n *= 6 chickens per group. The results were reported in SI units (mmo/L). The value for the control group was the average of 3 control groups. Values in columns not sharing a common symbol were significantly different at *P *< 0.05. For each figure, 'A' shows the raw values for each of the treatments and control, and 'B' shows the percent change compared to control. The groups 1 - 12 correspond to: 1. control diet; 2; δ-tocotrienol (δ-T3); 3. quercetin; 4. riboflavin; 5. (-) Corey lactone; 6. amiloride; 7. dexamethasone; 8. δ-T3 + quercetin; 9. δ-T3 + riboflavin; 10. δ-T3 + (-) Corey lactone; 11. δ-T3 + amiloride; 12. δ-T3 + dexamethasone.

**Figure 8:**
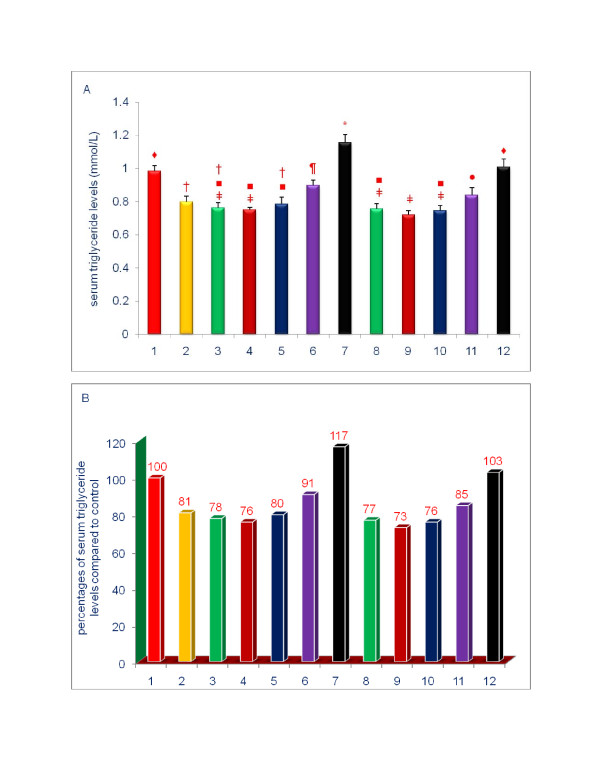
**Effects of dietary supplements on the serum levels of triglyceride of 5-week-old female chickens**. Chickens were fed for 4 weeks. The diets of groups 8 - 12 were supplemented with δ-tocotrienol (50 ppm). Data expressed as means ± SD, *n *= 6 chickens per group. The results were reported in SI units (mmo/L). The value for the control group was the average of 3 control groups. Values in columns not sharing a common symbol were significantly different at *P *< 0.05. For each figure, 'A' shows the raw values for each of the treatments and control, and 'B' shows the percent change compared to control. The groups 1 - 12 correspond to: 1. control diet; 2; δ-tocotrienol (δ-T3); 3. quercetin; 4. riboflavin; 5. (-) Corey lactone; 6. amiloride; 7. dexamethasone; 8. δ-T3 + quercetin; 9. δ-T3 + riboflavin; 10. δ-T3 + (-) Corey lactone; 11. δ-T3 + amiloride; 12. δ-T3 + dexamethasone.

The data shown in Figures 3, 4, 5, 6, 7, 8 are presented in two different formats. For each Figure., 'A' shows the raw values for each of the treatments and control, and 'B' shows the percent change compared to control.

Each of the experimental compounds significantly lowered serum TNF-α levels (Figure 3A, B). The decreases in serum levels of TNF-α with δ-tocotrienol (83%), quercetin (84%), riboflavin (81%), (-) Corey lactone (83%), amiloride (72%), and dexamethasone (61%), were significant (*P *< 0.01) as compared to control. Treatments combining δ-tocotrienol with the remaining compounds produced further reductions in serum levels of TNF-α with quercetin (5%), riboflavin (11%), (-) Corey lactone (5), amiloride (16%), and dexamethasone (13%) that were considered non-significant due to large standard deviation (>18% - 45%) in all groups (Figure 3B).

Serum NO levels were significantly lowered by treatments comprising only of δ-tocotrienol (45%), (-) Corey lactone (31%), and dexamethasone (67%) (*P *< 0.002) compared to control (Figure 4A, B). However, in comparing the results of combined supplementation vs. single compound supplementation, the addition of δ-tocotrienol to all compounds tested produced further significant (*P *< 0.001) reductions in serum NO levels with quercetin (25%), riboflavin (34%), (-) Corey lactone (18%), amiloride (32%), and dexamethasone (11%), as compared to their respective individual reduction (Figure 4B). It is important to note that the reduction of serum NO with δ-tocotrienol (45%) alone closely resembles the values of combined treatments of δ-tocotrienol with quercetin, riboflavin, (-) Corey lactone, amiloride (approximately 50%), except dexamethasone, which showed maximum reduction of serum NO (67%) alone versus combined treatment with δ-tocotrienol (78%; Figure 4B). These pronounced reductions of TNF-α and NO by δ-tocotrienol with a dose of 50 ppm may be due to maximal attenuation achieved with this dose, indicating that it is a very potent anti-inflammatory agent. The lower dose of δ-tocotrienol (10 or 20 ppm) in combination with quercetin, riboflavin or (-) Corey lactone may produce far better additive effects than observed in the present study. In summary, the pronounced reductions of serum TNF-α and NO levels by a near saturating concentration of δ-tocotrienol, likely, masked the responses to be gained with combined treatments.

### Effects of various compounds on serum levels of lipid parameters of 5-week-old female chickens

Serum total and LDL-cholesterol levels in chickens fed the δ-tocotrienol-supplemented diet were 77%, and 55% of control level, respectively (Figures 5, 6). These observations are consistent with results from numerous clinical trials [18,31-34,51]. All of the remaining compounds, with the exception of dexamethasone, significantly lowered serum total cholesterol levels, (-) Corey lactone by 31%, riboflavin by 22%, quercetin by 13% and amiloride by 9% (*P *< 0.05) compared to control (Figures 5, 6). Dexamethasone significantly increased serum level of total cholesterol to 220% (*P *< 0.001) of control level (Figure 5), an impact that was attenuated, by the addition of δ-tocotrienol. Although the combined treatments produced modest reductions (6% to 12%) in serum total cholesterol level when compared to reductions achieved with the individual compounds (Figure 5B), only the combination of δ-tocotrienol and (-) Corey lactone produced a greater reduction in serum total cholesterol levels than that achieved with δ-tocotrienol (Figure 5B).

Treatment-mediated effects on serum LDL-cholesterol levels were generally comparable to the effects of total cholesterol. With the exception of dexamethasone, all of the individual treatments yielded significantly lowered serum LDL-cholesterol levels (21% - 51%; *P *< 0.001) as compared to control (Figure 6). Dexamethasone significantly increased serum LDL-cholesterol level to 230% of control level (Figure 6B). The effect of dexamethasone was significantly attenuated by the addition of δ-tocotrienol (Figure 6B). Although combined treatments comprised of δ-tocotrienol and quercetin, riboflavin, (-) Corey lactone and amiloride yielded serum LDL-cholesterol levels lower than those attained with the individual agents, only the combination of δ-tocotrienol and (-) Corey lactone yielded a level significantly lower than that attained with δ-tocotrienol (Figure 6B).

Consistent with other reports [18,31-34,51], supplementation with δ-tocotrienol had no impact on HDL-cholesterol level (Figure 7). Quercetin, riboflavin and (-) Corey lactone lowered serum HDL-cholesterol levels modestly. Dexamethasone significantly increased serum HDL-cholesterol level to 218% of control (Figure 7B). The increase observed with dexamethasone alone was also attenuated by the addition of δ-tocotrienol, comparable to the findings with serum total cholesterol and LDL-cholesterol levels. Serum HDL-cholesterol levels in chickens treated with dexamethasone plus δ-tocotrienol were 182% of control level (Figure 7B). In comparing the results of combined dietary supplementation with single compound, the addition of δ-tocotrienol to all compounds tested, except amiloride, did not produce further reductions in serum HDL-cholesterol levels (Figure 7B). Individually, neither δ-tocotrienol nor amiloride reduced serum HDL-cholesterol levels; the combination of δ-tocotrienol plus amiloride, however, modestly reduced serum HDL-cholesterol level (Figure 7B).

With the exception of dexamethasone, all of the individual treatments yielded significantly lowered serum triglyceride levels (9% - 24%; *P *< 0.002) of control group (Figure 8). Dexamethasone significantly increased the serum triglyceride level by 17% (*P *< 0.001) of control (Figure 8B). This increase observed with dexamethasone was completely abrogated by the addition of δ-tocotrienol; serum triglyceride level in chickens treated with dexamethasone plus δ-tocotrienol was 103% of control level (Figure 8B). In comparing the results of combined treatments with single compound treatments, the addition of δ-tocotrienol to all compounds tested produced additional reductions in serum triglyceride levels (Figure 8B), only that achieved by combining δ-tocotrienol with amiloride was significant.

To summarize the results presented above, serum total cholesterol, LDL cholesterol, and triglyceride levels were generally decreased by diets supplemented with δ-tocotrienol, quercetin, riboflavin, or (-) Corey lactone, and minimally with amiloride. Treatments combining δ-tocotrienol with quercetin, riboflavin, (-) Corey lactone, or amiloride generally produced additional but mostly non-significant reductions. Dexamethasone increased serum total cholesterol, LDL-cholesterol, HDL-cholesterol, and triglyceride levels (Figures 5B, 8B). These increases in serum total and LDL-cholesterol levels with dexamethasone were attenuated by the addition of δ-tocotrienol.

### Microarray analyses of RNA of liver samples of 5-week-old female chickens

Cluster microarray data analyses of mRNA from pooled liver samples of each treatment using GeneSifter program provided valuable information comprising of 465 genes. Out of 465 genes, there were at least 62 genes whose expression was either up-regulated or down-regulated by δ-tocotrienol, quercetin, riboflavin, (-) Corey lactone, and dexamethasone. These 62 genes were categorized under inflammation, ageing, cardiovascular disease and cancer (Tables 3, 4). Out of 62 genes, only 39 genes were up-regulated (Table 3) and 23 down-regulated (Table 4) by these compounds. The expression of genes up-regulated by these compounds were associated with inflammation (9 genes), ageing (7 genes), cardiovascular disease (20 genes), and cancer (3 genes) as shown in Table 3. These compounds modulated the expression of a number of genes, such as: interferon 1 receptor, cytokine signaling, NFκB and ubiquitin protein lipase (inflammation), heat shock protein, RIKEN cDNA, ATPase, T cell receptor gamma (ageing), FAS, myosin, squalene epoxidase, NADH dehydrogenase, Prostaglandin D2 synthase, coagulation factor II (cardiovascular), and RAN, member of RAS oncogene family (cancer) as shown in Table 3.

**Table 3:** Microarray analyses of RNA of livers of chicken after treatment with various compounds.

		*Genes Up-regulated*						
#	**Genes**	**Control**	**δ-T3**	**Quer**	**Ribo**	**Co lac**	**Dexa**	**Description**

	**Inflammation**							

1	BX932265	0.15	1.30	3.69	1.57	2.78	1.83	Endothelin receptor type 2
2	BU449947	0.81	1.92	3.54	2.05	2.81	1.73	Endothelin receptor type B
3	AJ719289	1.10	2.65	3.12	2.10	2.75	2.07	Ubiquitin protein lipase E3C
4	NM_204124	1.12	2.40	3.12	3.09	3.11	2.03	Nuclear receptor subfamily 2, group C, member 1
5	AF082666	1.22	1.89	3.24	1.92	1.86	1.01	Interleukin 10 receptor, beta
6	CF256615	1.96	4.37	4.40	3.60	4.55	3.26	Interferon-gamma receptor alpha chain precursor
7	BU313956	2.41	2.75	4.64	2.99	3.24	3.21	Suppressor of cytokine signaling 1
8	AJ720966	2.54	4.72	4.95	4.29	4.66	4.09	Nuclear factor kappaB related to binding protein
9	NM_205485	3.35	4.40	5.91	5.06	5.28	5.10	Interferon 1 receptor, type 1

	**Ageing**							

10	CR523238	0.50	1.46	2.55	0.44	2.21	0.92	Palmitoyl-protein thioesterase 1
11	U22666	0.75	4.62	4.48	4.50	3.00	2.66	T cell receptor gamma
12	BX950427	0.97	3.37	4.68	2.44	4.25	2.29	RIKEN cDNA C230081A13
13	AF175433	1.96	4.00	5.05	3.80	3.50	2.20	T cell receptor delta chain (TCRD)
14	NM_205520	3.23	4.04	5.55	3.66	4.15	3.32	ATPase, Na+/K+ transporting, beta 1 polypeptide
15	AF387865	4.67	6.17	7.02	5.55	6.49	6.44	Heat shock protein 90 Da beta (Grp94), member 1
16	BX932093	6.04	8.07	8.34	7.24	8.47	8.01	PIT 54 protein

	**Cardiovascular**							

17	CR524241	0.46	3.47	6.00	2.39	6.61	6.57	Succinate-CoA ligase, GDP-forming, beta subunit
18	ENSGAL T4	0.48	1.73	2.55	1.81	1.91	1.06	Glycerol kinase 5
19	CR325238	0.50	1.46	2.55	0.44	2.21	0.92	Palmitoyl-protein thioesterase 1
20	ENSGAL T26	0.55	2.92	3.79	2.96	2.92	1.90	Cytosolic methionine-S-sulfoxide reductase
21	NM_205274	0.71	2.15	3.51	2.00	3.49	2.39	Myosin, heavy chain 11, smooth muscle
22	J04598	0.85	2.79	3.69	1.89	3.15	1.32	Collagen, type VI, alpha 1
23	ENSGAL T6	1.45	3.57	4.09	2.79	3.88	2.40	Plasminogen precursor
24	ENSGAL T5	1.53	2.99	4.09	3.17	3.78	3.29	Aldehyde dehydrogenase 9 family, member A1
25	BU235638	1.54	3.51	5.21	4.32	4.21	2.15	Serine hydroxymethyltransferase 1 (soluble)
26	BX930122	1.75	3.12	3.64	3.11	3.90	2.61	Aldehyde dehdrogenase 8 family member A1
27	ENSGAL T12	2.25	4.26	4.82	3.59	5.05	3.61	Glucose 6-phosphate translocase
28	AJ720577	2.37	4.78	6.86	4.80	6.74	5.97	NADH dehydrogenase (Ubiquinone) Fe-S protein 1
29	NM_204259	2.48	5.88	6.02	5.75	5.70	3.67	Prostaglandin D2 synthase 21 KDa
30	AJ720030	3.29	5.00	5.62	5.35	5.13	1.62	Squalene epoxidase
31	NM_205483	3.50	5.60	5.60	4.48	3.97	3.72	Lipoprotein
32	ENSGAL T10	3.83	5.08	5.43	5.49	5.89	3.28	Enoyl-coenzyme A, hydratase/3-hydroxyacyl coenz
33	NM_2051155	4.73	5.52	6.87	5.48	5.79	6.09	Fatty acid synthase
34	ENSGAL T20	5.09	5.86	7.01	6.32	7.11	5.70	Alcohol dehydrogenase 1C (class 1), g-polypeptide
35	NM_204605	5.83	7.57	7.98	7.05	7.68	6.68	Coagulation factor II (thrombin)
36	BX931521	6.36	8.27	8.94	8.12	8.43	8.63	Glutathione peroxide 3 precursor (GSHPx-3)

	**Cancer**							

37	AJ720735	0.18	1.76	2.70	0.90	2.87	1.37	Endoplasmic reticulum protein 29
38	BU117693	1.51	2.98	3.56	2.68	2.93	2.48	RAN, member RAS oncogene family
39	ENSGAL T6	2.60	5.10	5.88	4.53	5.81	4.98	Similar to cytochrome P450, family 2, subfamily W,

δ-T3 = δ-Tocotrienol; Quer. = Quercetin; Ribo = Riboflavin; (-) Co lac = (-) Corey lactone; Dexa = Dexamethasone

**Table 4:** Microarray analyses of RNA of livers of chickens after treatment with various compounds.

		*Genes down-regulated*						
**#**	**Genes**	**Control**	**δ-T3**	**Quer**	**Ribo**	**Co lac**	**Dexa**	**Description**

	**Inflammation**							

1	AJ719859	3.33	2.03	2.42	1.74	1.93	1.18	Proteasome (prosome, macropain) activator subunit 4
2	BX950555	4.01	4.11	4.38	3.78	3.38	1.64	Tumor necrosis factor superfamily, # 5-induced protein
3	BU397996	4.53	2.82	2.18	2.74	2.20	2.87	Protein Kinase

	**Ageing**							

4	CR353144	4.03	1.66	3.57	3.12	2.92	3.18	Nuclear DNA-binding protein
5	BU426315	4.50	2.20	2.42	2.14	1.70	1.33	Carnitine palmitoyltransferase 1A (liver)
6	CR523285	4.60	2.35	3.26	1.90	3.68	8.02	Finshed cDNA clone ChEST613j16
7	CD735693	6.05	4.25	5.44	4.66	3.68	3.39	Heat shock protein 25
8	BU123548	6.47	6.16	7.31	6.30	6.14	4.20	Finshed cDNA clone ChEST495e19
9	NM_205471	8.73	6.99	8.76	7.69	7.42	6.28	Phosphoenolpyruvate carboxykinase 1
10	CR523582	6.79	5.31	4.78	5.19	4.66	4.84	B-cell CLL/lymphoma 9

	**Cardiovascular**							

11	M64990	3.94	1.69	1.51	2.10	1.02	1.25	Prostaglandin-endoperoxide synthase 2
12	CR522967	4.42	3.41	2.18	3.20	2.53	3.67	KIAA1285 protein
13	ENSGAL T24	5.31	3.93	5.00	4.42	4.60	3.05	Aproteindipose differentiation-related
14	BX935098	5.79	4.45	5.41	4.35	4.52	3.09	Glutathione S-transferase theta 1
15	BU422942	5.84	4.14	3.65	4.09	3.79	4.41	Glycogen synthase kinase 3 beta
16	BU272340	6.58	5.63	4.23	5.47	5.39	5.81	Inositol hexaphosphate kinase 2

	**Cancer**							

17	AJ447153	2.19	0.83	2.06	1.56	1.21	0.01	Protein tyrosine phosphate, non-receptor type 2
18	BU463093	3.63	2.67	4.07	2.78	3.28	0.90	Amino acid transporter system A1
19	AL585963	3.95	2.15	1.74	1.20	2.07	1.67	RAS guanyl releasing protein 3
20	ENSGAL T0	4.24	3.18	1.62	2.42	1.85	2.97	Breast cancer-associated antigen BRCAA 1
21	BU131710	4.47	4.36	5.24	5.28	4.35	2.31	Isopentenyl-diphosphate delta isomerase 1
22	BU111042	6.54	5.46	4.39	4.81	4.98	5.95	Chrosome 6 open reading frame 111; SR rich protein
23	BU458470	7.84	5.78	7.08	6.96	5.90	5.90	Jun oncogene

δ-T3 = δ-Tocotrienol; Quer. = Quercetin; Ribo = Riboflavin; Co lac = (-) Corey lactone; Dexa = Dexamethasone

The expression of genes down-regulated by these compounds were associated with inflammation (9 genes), ageing (7 genes), cardiovascular disease (6 genes), and cancer (7 genes) (Table 4). Some of the important genes whose expression was modulated by these compounds included those of proteasome, protein kinase, tumor necrosis factor (inflammation), carnitine palmitoyltransferase 1A, nuclear DNA-binding protein (ageing), glycogen synthase kinase, glutathione S-transferase (cardiovascular), RAS guanyl releasing protein 3, and Jun oncogene (cancer) (Table 4). The Jun oncogene also plays important role in ageing.

The detailed analyses of genes whose expressions modulated by δ-tocotrienol, quercetin, riboflavin, (-) Corey lactone and dexamethasone, regulated differentially, were also selected (Tables 5, 6). Among these compounds, the first four were associated with lowering of serum lipids and latter, dexamethasone, associated as a lipid-elevating compound. Expression of 20 genes was up-regulated and 7 genes down-regulated by δ-tocotrienol, quercetin, riboflavin, (-) Corey lactone, except for a lipid elevating, dexamethasone (Table 5). Expression of 8 genes was up-regulated by first four compounds and down-regulated by dexamethasone, and 2 genes were down-regulated by first four compounds and up-regulated by dexamethasone (Table 6). Moreover, expression of 2 genes was up-regulated by dexamethasone, and not by the four lipid-lowering compounds and 1 gene was down-regulated by dexamethasone, and no effect was observed with the four lipid-lowering (δ-tocotrienol, quercetin, riboflavin, (-) Corey lactone) compounds (Table 6).

**Table 5:** Microarray analyses of RNA of livers of chickens after treatment with various compounds.

		*Genes up-regulated by the first four lipid-lowering compounds and lipid-raising dexamethasone*						
**#**	**Genes**	**Control**	**δ-T3**	**Quer**	**Ribo**	**Co lac**	**Dexa**	**Description**

1	BX275358	3.42	6.22	6.09	6.66	5.39	3.31	Putative ISG 12-2 protein
2	BU235638	1.54	3.51	5.21	4.32	4.21	2.15	Similar to serine hydroxymethyltransferase 1 (soluble)
3	ENSGL T6	3.98	6.05	6.93	5.53	7.15	4.59	UDP glucuronosyltransferase 1 family, polypeptide A10
4	CF256116	1.85	3.58	4.64	3.76	4.57	1.91	Phosphoribosyl pyrophosphate amidotransferase
5	NM_204858	-0.48	0.95	2.05	1.49	1.70	-0.13	Interferon (alpha, beta, and omega) receptor 2
6	AY534896	6.19	8.22	8.56	8.00	7.91	6.40	Gal 10
7	JO4598	0.85	2.79	3.69	1.89	3.15	1.32	Collagen, type IV, alpha 1
8	ENSGALT2	6.32	8.29	8.95	7.64	8.48	6.78	Similar to inter-alpha (globulin) inhibitor H3
9	ENSGALT27	0.84	3.10	3.18	2.23	2.50	0.87	Similar to KDEL (Lys-Asp-Glu-Leu) containing 1
10	M60069	6.14	8.06	6.71	7.57	7.88	6.00	Phosphoribosyl pyrophosphate amidotransferase
11	ENSGALT15	4.35	5.38	7.00	5.96	6.71	4.52	Similar to MGC 107895 protein
12	BX275222	0.23	2.43	2.64	1.24	1.59	0.19	Hypothetical protein LOC69044
13	ENSGALT27	4.80	6.48	7.13	5.83	6.84	5.06	Similar to thrombin-activatable fibrinosis inhibitor
14	BU219227	1.60	2.82	4.09	3.16	3.21	1.92	WD repeat domain 61
15	NM_205299	2.29	3.38	4.62	3.39	3.66	2.33	Dystrophin
16	ENSGALT26	2.10	3.46	4.27	3.23	3.67	2.66	Similar to methylmalonyl coenzyme A mutase
17	NM_205355	4.83	6.24	7.15	5.98	6.12	5.21	Ring finger protein 13
18	ENSGALT3	3.62	4.91	5.89	4.33	6.40	3.78	Similar to complement regulator factor H
19	CR290617	3.27	4.77	5.74	4.17	4.96	3.41	RER1 retention in endoplasmic reticulum 1 homolog
20	ENSGALT4	0.99	2.51	3.36	1.79	2.62	1.12	Similar to U1 snRNP-specific protein C
21	AF082666	1.22	1.89	3.24	1.92	1.86	1.01	Interleukin 10 receptor, beta

		***Genes down-regulated by the first four lipid-lowering compounds except dexamethasone***						

**#**	**Genes**	**Control**	**δ-T3**	**Quer**	**Ribo**	**Co lac**	**Dexa**	**Description**

1	ENSGALT20	2.01	1.14	-0.34	1.29	0.73	2.35	Similar to Trans-Golgi p230
2	BU270035	2.54	1.06	1.03	-0.08	1.25	2.59	Similar to Expressed soquence A1314180
3	BU208119	3.84	3.14	1.33	2.13	1.93	3.51	Similar to RIKEN cDNA D130059P03 gene
4	BU229724	5.44	3.99	2.96	3.49	3.57	5.55	Finshed cDNA, clone Chest295h22
5	ENSGALT9	1.84	-0.43	-0.23	0.75	-0.57	2.50	Similar to KIAA07 protein
6	BU305188	6.08	4.75	3.02	4.19	3.99	5.71	PCF11, cleavage and polyadenylation factor subunit
7	BU426927	3.76	2.44	-0.14	1.83	0.97	3.63	Triple functional domain (PTPRF interacting)

δ-T3 = δ-Tocotrienol; Quer. = Quercetin; Ribo = Riboflavin; Co lac = (-) Corey lactone; Dexa = Dexamethasone

**Table 6:** Microarray analyses of RNA of livers of chickens after treatment with various compounds.

		*Genes up-regulated by the first four lipid-lowering compounds,*						
		***and down-regulated by dexamethasone***						

**#**	**Genes**	**Control**	**δ-T3**	**Quer**	**Ribo**	**Co lac**	**Dexa**	**Description**

1	CR353609	4.47	7.23	7.13	6.67	7.12	3.95	Finished cDNA, clone CHEST110e20
2	AJ720605	3.07	5.39	5.65	5.23	5.04	2.66	Ornithine aminotransferase
3	BX265212	2.81	4.72	5.12	4.54	5.14	2.38	Similar to L-Kynurenine hydrrlase
4	CR405837	2.76	4.89	5.27	4.49	4.95	1.89	Finished cDNA, clone CHEST884a21
5	BU250153	3.57	5.25	5.97	5.13	5.77	3.27	Selenoprotein P, plasma, 1
6	AJ720030	3.29	5.00	5.62	5.35	5.13	1.62	Squalene epoxidase
7	ENSGALT10	2.97	4.76	5.19	4.00	4.83	3.86	Assembly = WASHUC1ǀchr = 20ǀstrand = forwardǀcdna
8	L07842	2.48	3.90	4.80	3.18	5.70	1.48	Antithrombin III

	**Genes**	***Genes down-regulated by the first four lipid-lowering compounds,***						

		***and up-regulated by dexamethasone***						

1	CR389189	4.60	2.35	3.26	1.90	3.68	8.02	Finshed cDNA, clone CHEST613j 16
2	NM204114	3.82	0.66	2.35	1.33	2.22	5.97	Deiodenase, iodothyronine, type II
3	NM_205155	4.73	5.52	6.87	5.48	5.79	9.09	Fatty acid synthase

	**Genes**	***Genes up-regulated only by dexamethasone***						

1	BU359098	0.84	1.08	1.04	1.25	0.88	3.69	BUB 1 uninhibited by benzimidazoles 1 homolog

	**Genes**	***Genes down-regulated only by dexamethasone***						

1	BU384885	2.11	3.48	4.56	2.90	3.43	1.23	Hypothetical LOC771662

δ-T3 = δ-Tocotrienol; Quer. = Quercetin; Ribo = Riboflavin; Co lac = (-) Corey lactone; Dexa = Dexamethasone

## Discussion

On average, the weight of chickens fed the control diet increased by 240 g during the four-week trial. In the current study, we found chickens fed a diet supplemented with δ-tocotrienol gained significantly less weight. This finding differs from results of earlier trials demonstrating that diet supplementation with δ-tocotrienol produced either no change, or an increase in weight gain [43,51]. Quercetin, amiloride, and dexamethasone yielded significantly lower weight gain, whereas riboflavin and (-) Corey lactone had no significant effect on weight gain. Interestingly, combined supplementation consisting of δ-tocotrienol plus either (-) Corey lactone or amiloride significantly increased weight gain; the combination of δ-tocotrienol and quercetin produced a weight gain equal to that of the control. Thus for (-) Corey lactone, amiloride, and quercetin, additional supplementation with δ-tocotrienol appeared to increase weight gains, as compared to dietary supplementation with each of these compounds alone. The addition of δ-tocotrienol to riboflavin did not improve weight gains compared to dietary supplementation with riboflavin alone. As reported elsewhere, dietary supplementation with dexamethasone markedly reduced weight gain compared to control [59,60], and this detrimental effect was enhanced by combining dexamethasone with δ-tocotrienol.

Histological examination demonstrated decreased inflammation in livers from chickens receiving individual treatments. Treatments combining δ-tocotrienol with either riboflavin, (-) Corey lactone, or amiloride yielded further decreases in hepatic inflammation and fatty infiltration. On the other hand δ-tocotrienol potentiated the toxic impact of dexamethasone. Dexamethasone toxicity, previously demonstrated in rats, was manifested by impaired growth, enlarged livers, and elevated serum total cholesterol and triglyceride levels [59-61].

All predictors of cardiovascular risk evaluated in this study were substantially decreased by all compounds with the exception of dexamethasone. Summarizing the overall effects of the individual compound on the five serum factors under consideration (TNF-α, NO, total cholesterol, LDL-cholesterol, and triglyceride) leads to the conclusion that the cumulative risk of atherosclerosis is reduced effectively by δ-tocotrienol, quercetin, riboflavin, and (-) Corey lactone.

Serum TNF-α levels of chickens receiving each of the compounds were uniformly lower than those recorded for chickens fed the control diet. Serum levels of TNF-α of chickens treated with δ-tocotrienol, quercetin, riboflavin, and (-) Corey lactone were reduced by approximately 80%. Serum TNF-α levels of chickens treated with dexamethasone and amiloride were reduced by approximately 41% and 70%, respectively. These findings are consistent with prior reports of the effects riboflavin [62-64], quercetin [5,9,10,65], δ-tocotrienol [15-17], amiloride [66] and dexamethasone [67]*in vitro *[9,10,15-17,21] and *in vivo *[62-64] on TNF-α level.

δ-Tocotrienol produced a 45% reduction in the serum NO level. Quercetin, riboflavin, (-) Corey lactone, amiloride, and dexamethasone reduced serum NO levels by 14%, 14%, 31%, 25%, and 67%, respectively. Quercetin has been reported to down-regulate inducible-NO synthase (iNOS) activity *in vitro *[5-10,68], and riboflavin, delivered by injection or infusion, inhibits NO synthesis and the concomitant increases in serum NO level in LPS-challenged mice [62-64]. Perhaps the most important result of the present study is the finding that combining δ-tocotrienol with other dietary supplements enhances suppression of serum NO and TNF-α levels, as compared to single compound supplementation; these results are particularly striking for NO level. Although, reduction of serum NO level with δ-tocotrienol alone closely resembles the reductions of δ-tocotrienol combined with other compounds, which may be due to maximal attenuations achieved with a dose of δ-tocotrienol (50 ppm) used in the present study. In our earlier dose-response study of δ-tocotrienol effects on the serum levels of total cholesterol and LDL-cholesterol in chickens, the maximum effective dose was found to be 200 ppm (51). Therefore, a minimum effective dose of 50 ppm was selected for the present study. This is the first report that describes the effects of δ-tocotrienol for avian pro-inflammatory markers (TNF-α and NO), which is consistent with findings that δ-tocotrienol is very potent anti-inflammatory compound as reported recently (47), and it is possible a lower dosage (10 or 20 ppm) of δ-tocotrienol may potentiate the anti-inflammatory actions of quercetin, riboflavin and (-) Corey lactone.

All treatments, except those involving dexamethasone, resulted in significantly lower serum total cholesterol, LDL-cholesterol and triglyceride levels. The effects of δ-tocotrienol and (-) Corey lactone on serum levels of total and LDL-cholesterol were significantly greater than those of the other compounds (Figures 5, 6, 8). Our current study appears to be the first observation of the cholesterol-lowering impact of (-) Corey lactone. δ-Tocotrienol is widely reported to effectively suppress HMG-CoA reductase activity and concomitantly lower serum total cholesterol and LDL-cholesterol levels [30-34,40-43,69,70]. Quercetin suppresses HMG-CoA reductase activity *in vitro *[71] and *in vivo *[72], and dietary intake of quercetin has been inversely correlated with total cholesterol and LDL-cholesterol levels in Japanese women [73]. Cholesterol levels in an elderly population were inversely correlated with serum riboflavin levels [74]. Thus, our findings that quercetin, and more potently, riboflavin lowered serum cholesterol levels are supported by the literature [72-74]. We also found that diet supplementation with amiloride, an FDA approved diuretic, was least effective in reducing total cholesterol level (9%). This finding is somewhat similar to results of a human study which showed that serum total cholesterol levels were not altered in subjects receiving amiloride concomitantly with hydrochlorothiazide [75]. Amiloride also failed to impact serum levels of cardioprotective HDL-cholesterol in the current study. HDL-cholesterol levels in chickens receiving quercetin, riboflavin or (-) Corey lactone were modestly reduced. Combining δ-tocotrienol with these compounds failed to raise HDL-cholesterol. On the other hand, the anti-inflammatory compound, dexamethasone, dramatically increased serum HDL-cholesterol level.

All compounds other than dexamethasone resulted in a significant lowering of serum triglyceride levels (Figure 8). The additive effect of combining δ-tocotrienol with another compound, with the exception of amiloride, on serum triglyceride was insignificant (Figure 8).

A recent report points to the superiority of the HDL-cholesterol/total cholesterol (HDL-chol/TC) ratio for monitoring cardiovascular risk compared to serum total cholesterol and LDL-cholesterol levels [76]. With the exceptions of those incorporating dexamethasone, all treatments resulted in higher HDL-chol/TC ratios than that calculated for the control group (0.51). Within the individual treatment group δ-tocotrienol (0.65; 127%), riboflavin (0.61, 120%), (-) Corey lactone (0.68, 133%) appear to be most effective in improving the ratios. The ratio calculated for δ-tocotrienol + quercetin (0.65, 127%) was modestly improved relative to that calculated for the quercetin group (0.56, 110%). These modest improvements were also observed by δ-tocotrienol combination with riboflavin (129%), and (-) Corey lactone (145%), without improving amiloride or dexamethasone ratios. In addition to the potential ability of quercetin, to reduce the cumulative serum risk factors for cardiovascular disease (total cholesterol, LDL-cholesterol, triglyceride, NO and TNF-α levels) we found exceptional risk-reducing value in two vitamins, δ-tocotrienol (a member of the vitamin E group; Figure 1), and riboflavin when fed at levels 4- and 10-times higher, respectively, than those normally found in commercial chicken feed. Based on the data presented in this study, it is reasonable to propose that supplementing a 2500 kcal diet with 40 mg of either vitamin (i.e. δ-tocotrienol or riboflavin) could be potentially beneficial in reducing cardiovascular disease risk in humans.

Microarray analyses of liver samples identified 62 genes whose expression was up-regulated (39 genes) or down-regulated (23 genes) by all compounds suggesting common impact on serum levels of TNF-α, NO, and lipid parameters. The most important up-regulated gene expression modulated by these compounds were associated with cytokine signaling, NFκB and ubiquitin protein lipase (inflammation), heat shock protein, RIKEN cDNA, T cell receptor gamma (ageing), FAS, myosin, squalene epoxidase, NADH dehydrogenase, Prostaglandin D (cardiovascular disease), and RAN, member RAS oncogene family (cancer). The down-regulated genes were associated with proteasome, tumor necrosis factor (inflammation), carnitine palmitoyltransferase1A (ageing), glycogen synthase kinase, glutathione S-transferase (cardiovascular disease), and Jun oncogene (cancer) as reported earlier [76-80]. The microarray array analyses further identified several other genes whose expression was differentially impacted by the compounds shown to lower serum lipid levels and dexamethasone, associated with markedly elevated serum lipids.

## Conclusions

Levels of serum markers for risk of inflammatory diseases (NO and TNF-α) are decreased by oral dietary treatments supplemented with naturally-occurring, synthetic or FDA approved compounds, δ-tocotrienol, quercetin, riboflavin, dexamethasone, (-) Corey lactone, and a diuretic, amiloride. When administered in combination with an apparently saturating dose of δ-tocotrienol, the risk-lowering impact of the remaining compounds was only modestly increased. Therefore, this finding suggests the possibility of a pronounced additive effect in the presence of a lower dose of the δ-tocotrienol. Serum NO levels increase during ageing process, as a consequence of a diminished regulation of the activation of NF-κB signaling [1,2]. These compounds may also block the activation of NF-κB and result in lowering serum TNF-α and NO levels. Confirming numerous reports, δ-tocotrienol, a post-transcriptional suppressor of HMG-CoA reductase activity, effectively lowered serum total cholesterol, LDL-cholesterol and triglyceride levels. Moreover, treatments incorporating the anti-inflammatory compounds, quercetin, riboflavin, and (-) Corey lactone alone and in combination with δ-tocotrienol resulted in lower serum total and LDL-cholesterol levels. However, anti-inflammatory dexamethasone increased serum lipid levels, actions partially attenuated in the presence of δ-tocotrienol. These novel findings demonstrate the potential value to be gained through investigations of the impact of various nutritional supplements, specifically the flavonoids, alone and in combination with δ-tocotrienol on predictors of age-associated diseases.

## Abbreviations

AP-1: activator protein-1; COX-2: cyclooxygenase-2; HMG-CoA: β-hydroxy-β-methylglutaryl coenzyme A; ICAM: intracellular adhesion molecule-1; IκB: inhibitory kappaB; IL-1α: interleukin-1α; IL-6: interleukin-6; IL-8: interleukin-8; iNOS: inducible nitric oxide synthase; LPS: lipopolysaccharide; MCP-1: macrophage chemoattractant protein-1; MIP-1α: macrophage inflammatory protein-1α; NF-κB: nuclear factor-kappaB; NO: nitric oxide; TNF-α: tumor necrosis factor-α; ROS: reactive oxygen species; VCAM: vascular cell adhesion molecule-1.

## Competing interests

The authors declare that they have no competing interests.

## Authors' contributions

All the authors were involved in the designing of the study. DMS completed the paper work of "University of Wisconsin-Madison Animal Care and Use Protocol Review Form". He also supervised the feeding of the chickens at Poultry research Laboratory, University of Wisconsin, Madison, WI. CJP edited the manuscript. JCR has checked the statistical analyses of all the data. All the authors have read and approved the final version.
